# An Overview of a Re-Emerging Disease in Italy: Bovine Tuberculosis Outbreaks in Cattle from MTBC-Free Territories

**DOI:** 10.3390/pathogens13110962

**Published:** 2024-11-05

**Authors:** Alice Giusti, Lorenzo Carbonetta, Filippo Fratini, Gabriele Spatola, Fiorenza Panerai, Stefano Pardini, Luca Cianti, Andrea Armani

**Affiliations:** 1 Department of Veterinary Sciences, University of Pisa, Viale delle Piagge, 2, 56124 Pisa, Italy; alice.giusti@unipi.it (A.G.); l.carbonetta@studenti.unipi.it (L.C.); g.spatola2@studenti.unipi.it (G.S.); 2 Azienda USL Toscana Centro, Zona Val di Nievole, Piazza S. Maria Nuova, 1, 50122 Firenze, Italy; fiorenza.panerai@uslcentro.toscana.it (F.P.); stefano.pardini@uslcentro.toscana.it (S.P.); luca.cianti@uslcentro.toscana.it (L.C.)

**Keywords:** *Mycobacterium bovis*, zoonotic disease, eradication programmes, regional plans, surveillance, competent authority, livestock–wildlife interaction

## Abstract

Bovine tuberculosis (bTB) is a zoonotic disease with consequences for public health as well as the economy. In the EU, compulsory eradication programmes have been applied, and most territories in Italy have been reported as disease-free (FTs). However, outbreaks (OBs), i.e., an officially confirmed occurrence of bTB in one or more animals in an establishment, have continued to be reported. In this study we provide an overview of bTB in terms of OB numbers in cattle from Italian FTs. Legislative sources were collected to find the FTs, the relevant declaration of free status year (FSY), and regional control and surveillance plans. Then, descriptive and statistical analyses were applied to the collected OBs. A total of 12 regions and 19 provinces were declared FTs in the 20 years from 2003 to 2023. Differences in regional plans were observed with respect to the percentages of herds that were annually controlled (control frequency). Overall, 370 OBs were recorded. A non-statistically significant decrease in the OB incidence rate after the FSY was declared. However, a notable increase in OBs detected at slaughterhouses after the FSY suggests that control systems (serological tests) at the herd level are not completely effective. Differences in the herds’ control frequencies among FTs seem to not have had a significant influence on the observed OB number. The Tuscany region was the most affected FT based on the OB numbers after the FSY (especially in the last year). Epidemiologically relevant primary determinants seem to be the farming system (semi-extensive and adjacent herds) and the cattle movements from positive incidence areas (trade and animal fairs). The role of wild boars in the disease maintenance cannot be excluded. The results of this study stress the need to revise bTB eradication and surveillance plans based on risk analysis.

## 1. Introduction

Mammalian tuberculosis is a globally distributed chronic bacterial disease of animals and humans caused by members of the *Mycobacterium tuberculosis* complex (MTBC). Within the MTBC, important variants include *M. bovis*, *M. caprae*, and *M. tuberculosis* [1].

Bovine tuberculosis (bTB) is caused mainly by *M. bovis*, which is considered to have the widest host range [2]. It can be transmitted by a number of routes, especially inhalation or contact with the excreta of infected animals [3], as well as the ingestion of contaminated food and water [4]. Cattle, the main host, can become infectious long before they exhibit clinical signs [5]. In this species, the primary broncho-pneumonic infection may remain localised or progress slowly for a considerable amount of time, eventually leading to generalised lesions [6].

Other than cattle, *M. bovis* can infect a wide range of domestic and wild animal species, including humans [7,8]. The presence of *M. bovis* in wildlife usually originates from infected livestock herds but may thereafter spread between wildlife species and back to livestock [9].

Risk factors for zoonotic tuberculosis include high human–animal density, consumption of unpasteurised milk and milk products, close and frequent physical contact between humans and infected animals, and inadequate disease control measures [10,11,12]. Slaughterhouses are also a major source of occupational exposure to bTB along the entire meat production line [13,14]. The highest rates of zoonotic bTB incidence have been reported in Africa and Southeast Asia, while it is among the lowest in the world in the European region [11]. In 2022, there were 130 confirmed cases of human tuberculosis due to *M. bovis* or *M. caprae*, corresponding to a European Union (EU) notification rate of 0.03 cases per 100,000 people. This resulted in a notification increase of 13.2% compared with 2021 [15]. The highest number of EU notifications was reported in Member States not officially free from bTB [16]. The majority of cases in humans (68.5%) were of EU origin (native cases and/or cases originating from other Member States) [15].

Basically, bTB has a marked economic importance because of the loss in productivity due to morbidity and mortality, in addition to the potential zoonotic risk [17,18]. The surveillance costs also have immense economic importance [17]. At the EU level, bTB is listed in Regulation (EU) 2016/429 (“Animal Health Law”—AHL) [19] among the transmissible animal diseases which pose a risk to animal or public health in the EU and that must be notified to the Competent Authority (CA). The zones with non-MTBC-free status, belonging to 20 Member States, account for 60.4% of the whole EU cattle herd population [20]. Member States which are not MTBC-free throughout their territory or in zones thereof, should establish a programme for bTB eradication. The programme must be carried out in the cattle populations covering the relevant parts of their territory or the relevant zones thereof (i.e., a defined compulsory eradication programme). It must be applied until the conditions of MTBC-free status in the territory of the concerned Member State or zone are satisfied [19]. At least 99.9% of bovine herds should achieve official MTBC-free status to be considered MTBC-free. The MTBC-free status may only be granted to a herd if: i) during the past 12 months there has been no confirmed case of infection with MTBC in bovine animals kept in the establishment, and ii) the bovine animals more than 6 weeks of age present in the establishment at the time of testing or sampling have tested negative to immunological tests on two occasions, as detailed in the legislative text [21]. Other details for granting MTBC-free status, the requirements for its maintenance, as well as the conditions for its suspension/restoring and withdrawal/regaining, are laid down in the Commission Delegated Regulation (EU) 2020/689 [21]. Eradication programmes established in EU Member States typically consist of test-and-slaughter schemes [7]. The tuberculin skin test, evaluating a delayed-type hypersensitivity reaction in sensitised animals, is reported as the diagnostic test by EU legislation [21]. In addition, the gamma-interferon assay may be used in combination with the tuberculin skin test to increase the overall sensitivity and specificity of herd testing [21]. In the case of positive-tested cattle, the MTBC-free status of the establishment must be suspended [21]. bTB surveillance is completed at the slaughterhouse. Positive cattle are in fact compulsorily slaughtered on different days from healthy cattle (the so-called “deferred slaughter”), or on the same day but after the healthy cattle have been slaughtered. Subjected to postmortem examination, all animal carcasses are examined for typical macroscopic lesions, consisting of well-circumscribed (often encapsulated) foci of granulomatous inflammation with central necrosis or mineralisation [22,23].

Macroscopic lesions are frequently detected in the lymph nodes of the head and thorax (i.e., retro-pharyngeal, bronchial, and mediastinal), involving the lung parenchyma in 10–30% of cases, while generalised distribution of gross lesions is sporadically observed [23,24]. The CA defines a suspected case of bTB when: (a) clinical or postmortem examinations conclude that clinical sign(s), or postmortem lesion(s) are indicative of bTB; (b) result(s) from diagnostic methods indicate the likely presence of bTB in a sample from one or more cattle; or (c) an epidemiological link with a confirmed case has been established. The bTB case is confirmed if the disease agent has been isolated [21].

After disease confirmation, the MTBC-free status may be regained only if all bovine animals more than 6 weeks of age present in the establishment have tested negative in two immunological tests as follows: (i) the first test must be carried out on bovine animals or samples taken from bovine animals not earlier than 2 months after the removal of the last confirmed case and of the last animal that tested positive in an immunological test; and (ii) the second test must be carried out on bovine animals or on samples taken from bovine animals not earlier than 2 months and not later than 12 months following the date of the first test [21]; moreover, at least one of the conditions listed in the Part II, Chapter 1, Section 4b should be applied [21].

In the last 10 years (2013–2022), the annual number of positive cattle herds in EU zones under the eradication programme has decreased by 46.3%. However, this decrease is mainly attributable to the withdrawal of the United Kingdom from the EU in 2020 [15]. Indeed, the United Kingdom (including Northern Ireland) (12.2%), together with Ireland (4.6%) and Spain (2.5% in zones under an eradication programme), were the only countries that reported a prevalence higher than 1% [15]. In Italy, legislation governing the eradication programmes is essentially represented by the law of 9 June 1964, n. 615 [25], the Ministerial Decree of 15 December 1995, n. 592 [26], the Ministerial Ordinance of 28 May 2015 [27] and subsequent extensions (the last dated 15 June 2023), and the Legislative Decree of 5 August 2022, n. 136 [28]. Eradication programmes are developed annually, with the last reported in the Annex II of the decree dated 2 May 2024 [29], and implemented in both non-MTBC-free and MTBC-free territories. In the first case, control actions are to be carried out comprehensively on 100% of the bovine animals (>6 weeks of age) and herds through tuberculin skin testing of each individual animal. Animals that test positive are culled within 15 days. In the “MTBC-free” territories, surveillance is carried out with actions that include the testing of all bovine animals (>6 weeks of age) in a percentage of herds chosen according to current regulations and specific for each territory (regional plans) (Annex II of [29]). If lesions referrable to bTB are found during postmortem examinations, samples of the organs are sent to the Experimental Zooprophylactic Institute for the analysis. The National Reference Centre for *M. bovis* Tuberculosis (NRC) was established at the Experimental Zooprophylactic Institute of Lombardy and Emilia-Romagna. In addition, when lesions are found during routine slaughter, all animals >6 weeks of age from the originating establishment are tested using the tuberculin or gamma interferon tests. Within two days of the suspicion, the local CA initiates an epidemiological investigation to determine the source of the infection. In case it concludes that wildlife could be a possible source of infection, the CA implements a monitoring programme for MTBC in the involved wildlife species [29]. Thanks to this, bTB is now eradicated in most parts of the nation. However, bTB cases in cattle from MTBC-free territories continue to be reported [16,30].

In this study, bTB outbreaks in cattle from Italian MTBC-free territories (region or province) were collected and analysed with the aim to portray their occurrence before and after the year of MTBC-free status declaration in the respective territory. Outbreak occurrences were also discussed in light of the provisions outlined in the regional plans for the control and surveillance of MTBC-free territories, to evaluate the suitability of the control systems. Finally, the possible epidemiologically relevant primary determinants for the bTB outbreaks observed in the Tuscany region, one of the regions with the most bTB outbreaks, were considered.

## 2. Materials and Methods

### 2.1. Data Collection

#### 2.1.1. Legislative Sources

All of the Italian MTBC-free territories (FTs) (regions and/or provinces) and the relative year of MTBC-free status declaration (FSY) were found by consulting the EU documents in EUR-lex (https://eur-lex.europa.eu/ accessed on 14 March 2024). Starting from the most recent Commission Implementing Regulation (EU) 2023/1071 [31], all of the regulations regarding the approval or withdrawal of the disease-free status of certain Member States were searched backwards to identify the FSY of each FT. In addition, the regional plans for the control and surveillance of bTB of each individual FT were searched online for information on dispositions of cattle movements and the control frequency, projected as the percentage of herds that must be annually tested serologically to maintain the MTBC-free status in the FT. Only for the last considered year (2023), the number of controlled herds for each FT was approximately calculated based on the number of herds reported in the database of the National Informative Veterinary System (NIVS; https://www.vetinfo.it/j6_statistiche/#/report-pbi/1, accessed on 3 June 2024).

##### 2.1.2. bTB Outbreaks in Italian FTs

The official database contained in the National Veterinary Epidemiological Bulletin (NVEB; https://www.izs.it/BENV_NEW, accessed on 14 March 2024) was used for data collection. For each FT (regions and provinces) detailed in Section 2.1.1, all of the reported bTB outbreaks (OB) in bovine cattle until 2023 (last complete year) were collected. The number of infected cattle for each reported OB is not available in the NVEB. Therefore, in this study, OB means the officially confirmed occurrence of the disease in one or more animals in an establishment or other place where animals are stocked or located, as defined by the AHL [19]. In addition, the following data were retrieved from the NVEB for each OB: (i) if it was detected at herd level on the field (clinically or serologically) or at the slaughterhouse by the presence of macroscopic lesion/s during postmortem examination; (ii) if it was still open or closed. Specifically, a closed OB is defined as when the conditions for the establishment’s MTBC-free status (also described in the Introduction section) have been addressed [21].

### 2.2. Descriptive Analysis

For each FT (regions or provinces) detailed in Section 2.1.1, the data collected from NVEB (Section 2.1.2) were used to obtain the following information (overall and divided for each FT): (i) overall number of OB; (ii) number of OBs after the FSY; (iii) number of open and closed OBs in the years after the FSY; (iv) percentages of OBs detected at herd level on the field (clinically or serologically detected) and at the slaughterhouse during macroscopic examination of carcasses (before and after the FSY). Data were stratified by region and, when necessary, Italy was conventionally divided in the NUTS-1 areas (http://www.statoids.com/uit.html, accessed on 14 March 2024): northwest (NW) (Lombardy, Liguria, Piedmont, Valle d’Aosta regions), northeast (NE) (Emilia-Romagna, Friuli-Venezia Giulia, Trentino-Alto Adige, Veneto regions), central (Lazio, Marche, Tuscany, Umbria regions), and south (Abruzzo, Apulia, Basilicata, Campania, Molise regions), as well as the islands (Sardinia and Sicily). In the case of Tuscany, speculation on the possible epidemiologically relevant primary OB determinants is provided in light of the expertise of the authors.

### 2.3. Statistical Analysis

Statistical analyses were performed using Python 3.12. To compare the OB incidence rate (number of OBs/year) before and after each FT was declared MTBC-free, a Poisson regression model was applied. The model was adjusted for the varying lengths of the observation periods across different FTs. It was assumed the number of OBs reported in each FT was a “dependent variable”, and a binary indicator representing the period (0 = before the FSY, 1 = after the FSY) was an “independent variable”. The natural logarithm of the number of years of observation before and after the FSY was included as an offset in the model to account for the differences in the length of observation periods across FTs. The model was fitted using the Generalized Linear Model (GLM) framework with a Poisson distribution and a log link function. The statistical significance of the independent variable was assessed to determine if there was a significant difference in the OB incidence rate before and after the FSY was achieved. The 95% confidence intervals (*CI*) were calculated as:

CI=β±1.96×SE

where β is the estimated regression coefficient and *SE* the associated standard error derived from the Poisson model, based on the variance of the estimated coefficients.

To determine if there was a significant association between the OB detection site (herds or slaughterhouse) and the time period (before or after the FSY), a chi-squared test of independence was performed. The observed OB frequencies were tabulated in a 2 × 2 contingency table. The expected frequencies were calculated based on the assumption of independence between the two variables. The chi-square statistic was computed, and the associated *p*-value was obtained.

Finally, based on the number of controlled herds for each FT (Section 2.1.1), we performed correlation analyses using both Pearson and Spearman correlation coefficients to assess the relationships between the numbers of herds tested and the numbers of OBs (only those detected at the herd level). The Pearson correlation coefficient was used to evaluate linear relationships, while the Spearman correlation coefficient was employed to assess monotonic relationships.

The 95% *CI* were calculated as:

$$CI={r\pm 1.96\times SE}_{r}$$

where r is the estimated correlation coefficient and ${SE}_{r}$ the associated standard error calculated as ${SE}_{r}=\frac{{1-r}^{2}}{\sqrt{n-2}}$.

Differences among groups were considered significant for *p*-values ≤ 0.05.

## 3. Results and Discussion

### 3.1. Investigated FTs

**Figure 1:**
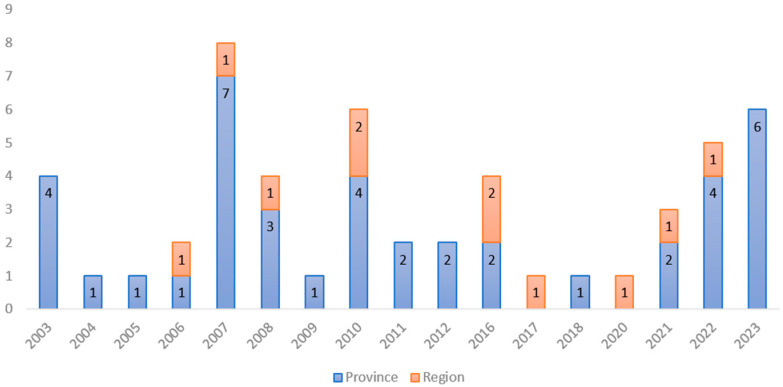
Number of Italian territories (regions and provinces) that acquired the MTBC-free status for each considered year.

Overall, 19 EU regulations regarding the approval or withdrawal of MTBC-free status in Italian territories were found, issued from 2003 to 2023, with a four-year gap in 2012–2015 (Table S1). The Italian territories can be assumed as the zones defined in the AHL [19] as “areas of a Member State [..] with a precise geographical delimitation, containing an animal subpopulation with a distinct health status with respect to a specific disease or specific diseases subject to appropriate surveillance, disease control and biosecurity measures”, and coincide with regions and provinces. The Trentino-Alto Adige region (divided in the autonomous provinces of Trento and Bolzano) was declared as an FT before 2003 [32]; therefore, a total of 12 regions are now FTs. By consulting these regulations, 11 regions and 41 provinces had acquired the MTBC-free status in the aforementioned period. Overall, the highest number of territories acquired the MTBC-free status in 2007 (one region and seven provinces), with an increasing trend in the last two years (2022, 2023), with five and six FT added, respectively (one region and ten provinces) (Figure 1).

The FT regions are all (100%) those from the NE (*n* = 4) and NW (*n* = 4), two of the four (50%) central regions, and two of the five (40%) southern regions (Table 1).

Four NE regions acquired their MTBC-free status earlier (before 2003 to 2008) than the other regions. In contrast, the two southern regions acquired their free status in the last two years (2021–2022). In general, southern FTs (regions and provinces) acquired their status more recently than the NE, NW, and Central ones. Indeed, in the southern regions, bTB eradication is more difficult, probably due to the type of farming that is based on mountain grazing and transhumance [22]. However, based on the prevalence reported in the 2024 national eradication programme [15], all of the Italian regions are predicted to be MTBC-free in 2030. Sicily is currently the only unique region still completely lacking FTs.

**Figure 2:**
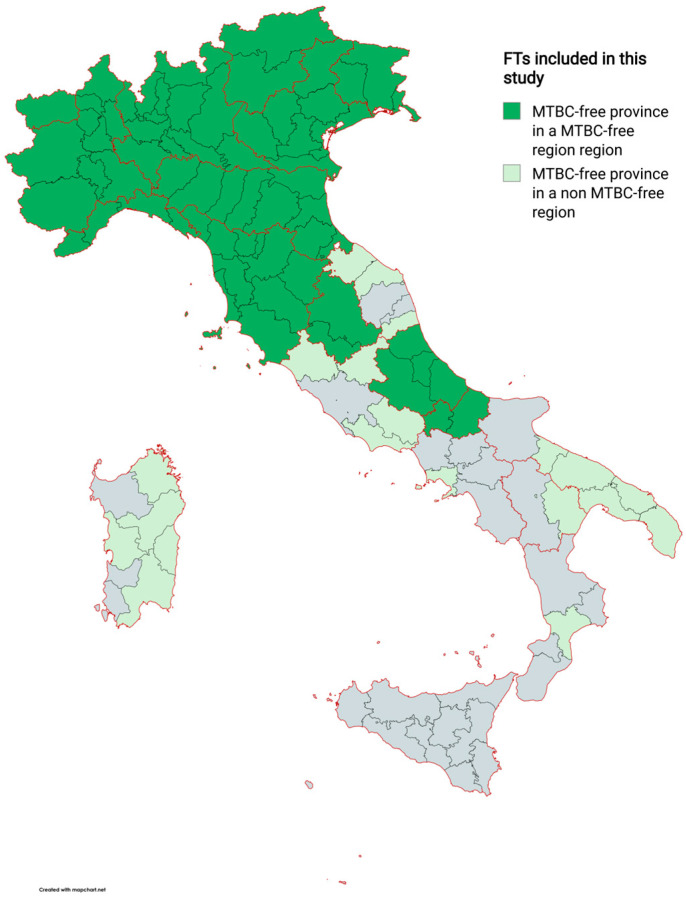
Geographic representation of MTBC-free territories (regions and provinces) (FTs) included in this study. The Sardinian provinces are those reported in the recent reform of the provinces made in 2023 [33], although FTs included in this study were the five provinces in force from 2016 to 2021. Image created with mapchart.net.

Six of the twelve FT regions, namely Trentino-Alto Adige, Abruzzo, Lombardy, Piedmont, Tuscany, and Veneto, did not acquire their MTBC-free status directly, but after the free status acquisition of one or more of their province/s in the previous years (Table 1). By removing these provinces from the overall count, 23 provinces have been declared as FTs. It should be also noted that in the Sardinia region, a recent reform of the provinces was made in 2023 [33]. However, considering that the reform should have been completed in 2024, the data in this study are those related to the division in the five provinces in force from 2016 to 2021. Due to this, the overall number of FT provinces drops further to 19. The FTs finally analysed (MTBC-free region and province) are detailed geographically in Figure 2.

### 3.2. FT Regional Plans for the Control and Surveillance of bTB

The regional plans of eleven of the twelve FT regions were retrieved, but the plans of the Trentino-Alto Adige region were not available online. The plans of the FT provinces were included in the regional plans of the respective region but only the plans of the provinces from the Lazio and Marche regions were found. Indeed, since the FSY of the other provinces were more recent (from 2021 to 2023), only the plans referring to previous years (thus concerning non-MTBC-free zones) are currently available online.

All of the regional plans state that the introduction of cattle in the territory is allowed only if the animals come from MTBC-free establishments. This actually reflects the national legislation, stating that fattening farms must be comprised of animals originating from MTBC-free establishments, subjected to favourable diagnostic checks within the 30 days preceding their introduction, if the animals are older than six weeks [27,34].

In contrast, the control frequency reported in the regional plan is specific for each FT, the choice of diagnostic methods is determined by the local CA, and it is influenced by the prevalence and incidence rates of infection or the epidemiological situation of the herd (Annex II of [29]). Eight of the eleven regional plans reported the percentage of herds that must be annually controlled (Table 2): 33% herds/year (100% in 3 years) in Friuli Venezia-Giulia; 25% herds/year (100% in 4 years) must be controlled in Lombardy, Umbria, and Valle d’Aosta; 20% herds/year (100% in 5 years) in Liguria, Tuscany, and Veneto; the regional plan of Abruzzo, FT only since 2022, reported that 100% herds/year must be controlled for the first two years after the FSY. Then, if the average annual percentage of infected herds decreases over the years, the percentages of herds that must be checked each year will decrease in turn.

The regional plans of Piedmont and Molise did not report any information. The regional plan of Emilia-Romagna is unique, not reporting the percentage of herds that must be annually controlled but only that all of the animals > 24 months must be checked every 3 years. According to the regional plans of the FT provinces, 50% herds/year must be controlled in Ancona, Ascoli Piceno, and Pesaro-Urbino (Marche region) and 25% in Rieti, Frosinone, and Viterbo (Lazio region) (Table 2). According to data reported in the NIVS database, results on the number of controlled herds for each FT in 2023 were used for the statistical analysis and are presented below in Section 3.3.2.

### 3.3. Descriptive and Statistical Analyses

#### 3.3.1. OB Number and Type

Data contained in the NVEB originate from the Informative System of Animal Disease (*Sistema Informativo Malattie Animali*—SIMAN), a centralised system for collecting, disseminating, and automatically analysing information on outbreaks of all notifiable animal diseases. Through secure access, CA can enter data and information regarding suspected and confirmed positive cases. Once recorded, this information can be transmitted to international organisations, consulted, and evaluated within the context of epidemiological studies on animal diseases. In FTs, the registration in SIMAN of the OB confirmation, accompanied by the related epidemiological investigation, entails immediate notification to the EU through the Animal Disease Notification System (ADNS) [27]. Although the NVEB database was available from 2004, data only began to be updated in real-time from 2010. This aspect must therefore be considered for data interpretation, meaning that OB numbers for the years before 2010 could be under-estimated.

**Figure 3:**
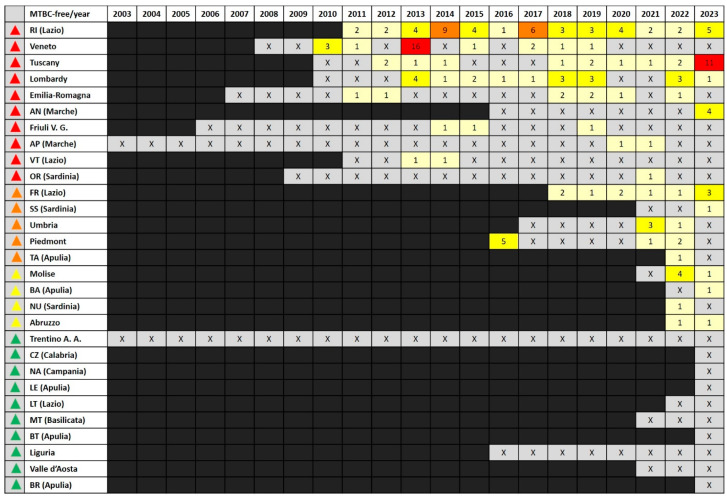
Bovine tuberculosis outbreaks (OB) in each bTB-free territory (FT) after the year of free status declaration (FSY). Red triangle: 100% of OBs after the FSY; orange triangle: 25% to 55.5% of OBs after the FSY; yellow triangle: 7.1% to 14.3% of OBs after the FSY; green triangle: no OBs after the FSY. AN: Ancona; AP: Ascoli Piceno; BA: Bari; BR: Brindisi; BT: Barletta-Andria-Trani; CZ: Catanzaro; FR: Frosinone; LE: Lecce; LT: Latina; MT: Matera; NA: Napoli; NU: Nuoro; OR: Oristano; RI: Rieti; SS: Sud Sardegna; TA: Taranto; VT: Viterbo; X: no OBs; light yellow: 1–2 OBs; yellow; 3–5 OBs; orange: 6–10 OBs; red: more than 10 OBs.

Overall, 370 OBs were recorded for the FTs. An average of 12.3 OB/FT (SD 12.5) was observed. No OBs were found in the Trentino-Alto Adige region or in two provinces (Cagliari and Pesaro-Urbino). The overall highest number of OBs (*n* = 47) was observed in the province of Rieti (Lazio), followed by Catanzaro (Calabria) (*n* = 36) and the Molise (*n* = 35) and Abruzzo (*n* = 28) regions (Table S2). These data should be considered poorly informative with respect to the actual status of the FT, as it also includes OBs detected before the FSY. In contrast, almost 50% (*n* = 166; 44.9%) of these OBs were dated after the FSY of the respective FT (see Table S2). In detail, 10 FTs (five regions and five provinces) counted 100% of their OBs after the FSY, and nine FTs (four regions and five provinces) counted 7.1–55.5% of their OBs after the FSY (Table S2). In the first case, all of the FTs were considered MTBC-free for ≥13 years, except for the province of Ancona (Marche) that was declared MTBC-free in 2015. In the second case, all of the territories are considered MTBC-free for ≤8 years (Figure 3).

Similarly, most of the FTs in which no OB had been reported after the FSY are considered MTBC-free for ≤3 years, except for Liguria (8 years). Therefore, it is clear that FTs showing more OBs after the FSY were mostly those that were declared MTBC-free for more years. Consequently, also considering that data reported in NVEB before 2010 are less reliable, these findings were quite predictable. The number of OBs recorded after the FSY per year ranged from 1 to 16. In most cases (71.8%), one or two OBs per year were observed, in consecutive years or interspersed by year/s with no OBs (Figure 3). Sixteen and eleven OBs were observed in Veneto region in 2013 and in the Tuscany region in 2023, respectively (Figure 3). However, while no OBs were reported since 2020 for Veneto and Tuscany, with 50% of the OBs (*n* = 11) only in the last year, and 77% (*n* = 17) in the last five years, were found to be particularly affected by the disease (see Section 3.4).

By applying the Poisson regression analysis, the coefficient for the period after the FSY was −0.0580 (*p*-value = 0.734), with a 95% *CI* of −0.291, +0.175. Therefore, the slight decrease in the OB incidence rate observed after the FSY was not statistically significant, suggesting that no measurable reduction in the OB incidence rate was observed.

Cases of OBs in Italian FTs (after the FSY) are reported in the literature. Amato et al. [30] described a case of bTB in a dairy cattle farm of the Veneto region in 2015. Magnani et al. [16] reported bTB cases in three dairy cattle herds in the Emilia-Romagna region in 2018 and 2019. In both of these studies, the causative agent was identified as *M. caprae* [16,30]. The same agent was first isolated from cattle reared in the Trento province in 2006 and in the Emilia-Romagna region in 2011, as well as in other regions of northern Italy (Lombardy and Veneto) [16]. Overall, our results suggest that, despite the measures implemented to keep the disease under control, bTB continues to be endemic. Therefore, the constant and careful application of the eradication programmes is crucial.

Most of the OBs reported after the FSY (158 of 166; 95.2%) were registered as closed. The remaining eight were still open, all dated after 2022 (included), and they were distributed as follows: four (one in 2022 and three in 2023) in Tuscany, two in Frosinone (Lazio) (in 2023), one in Lombardy (in 2023), and one in Abruzzo (in 2022). Particularly affected was Tuscany, associated with more cases of open OBs (Section 3.4).

#### 3.3.2. OB Detection Sites and Correlation with FT Control Frequency

Percentages of OBs detected at herd level in the field (clinically or serologically detected) and at the slaughterhouse (during macroscopic examination of carcasses) were distributed as follows: of the 204 OBs reported in all of the FTs before the FSY, 174 (85.2%) were detected at the herd level (10 clinically and 96 serologically) and 30 (14.7%) at the slaughterhouse; of the 166 OBs reported after the FSY, 106 (63.9%) were detected at the herd level (5 clinically and 169 serologically) and 60 (36.1%) at the slaughterhouse. At the herd level, detections based on serological tests were generally more numerous in both the considered periods (9× and 38× higher, respectively). This was expected, as infection in cattle is often subclinical. The results obtained from the chi-squared test (*p* value < 0.00001) showed that OB distribution was significantly altered before and after the FSY, with a notable decrease in OBs detected at the herd level and a corresponding increase in OBs detected at the slaughterhouses. Note, however, that OBs detected at the slaughterhouse are not necessarily linked to the FT in which the animal was slaughtered.

Lower percentages of OB detection at the herd level in favour of the slaughterhouse could be read as a negative event for the surveillance and eradication plans, since it implies that infected animals have evaded the control systems during breeding (serological tests), and they have been sent to routine slaughter. On the other hand, this does confirm that postmortem examination plays a primary role in detecting the disease in tuberculin-negative animals, being considered a cost-effective procedure in bTB surveillance [35,36]. According to EU legislation, even in the case of non-suspicious animals, the postmortem examination at the slaughterhouse should pay particular attention to the detection of this disease [37]. The more recent EU legislation, based on the scientific opinion of the European Food Safety Authority (EFSA) [38], imposes the omission of palpation and incision during the postmortem examination of bovine animals subjected to routine slaughter to reduce biological hazards. However, palpations and incisions are still considered necessary by the same legislation to survey the occurrence of bTB, except for animals less than 20 months old if reared under specific conditions [37].

These outcomes prove that it is crucial to continue performing targeted postmortem examinations, even in those subjects that, from pathogenic and epidemiological perspectives, would be considered low risk for infection. Scoppetta and Mancini [39] described a bTB case in a calf (10 months old) raised in fixed-stall fattening phases in Abruzzo, which tested negative after the tuberculin test. Similarly, in Emilia-Romagna, tubercular lesions during the postmortem examination of a slaughtered dairy cow were detected by the official veterinarians. The animal was tested in the herd using a tuberculin test two years previously, with a negative result, as did all of the other cattle of the herd [16]. As reported by Guardone et al. [40], analysis of data from postmortem examinations at the slaughterhouse is a useful tool to discover the trend of the main diseases over time, the results of control efforts, and to monitor compliance with animal welfare standards.

It should be highlighted, however, that lesions are not always detectable as they are sometimes invisible to the naked eye, limiting the accuracy of this approach [36]. Indeed, the primary complex is most frequently located in the lower parts of the respiratory tract and 70% of these lesions may only be found during careful examination and dissection of the lungs into thin sections [23]. Also, not all infected cattle have lesions at the time of slaughter, since the presence of bTB lesions is directly related to the interplay between the host’s defence mechanisms and mycobacterial virulence factors [23]. In addition, differences across slaughterhouses in lesion detection is variable due to internal factors such as line speed, light, and inspector performance efficiency, which should all be considered [36]. The use of the simplified inspection method proposed by the EU legislation, excluding palpation and incision in young cattle (<8 months), was hypothesised as decreasing the chance of finding lesions and could delay the success of an eradication programme [35].

The statistical analysis revealed weak correlations between the number of herds tested (control frequency) and the number of OBs detected at the herd level in 2023 (Table 2), with a Pearson’s coefficient of −0.0821 (95% *CI* −0.644, +0.480; *p*-value 0.780) and a Spearman’s coefficient of 0.2253 (95% *CI* −0.312, +0.763), indicating no significant relationship. Therefore, other factors not considered in this analysis may have a more significant influence on the observed OB number.

Overall, the resurgence of bTB in some FTs could be linked to a combination of factors, such as proximity with wildlife and/or other herds, and cattle movements. Quantifying the relative role of the potential factors on bTB occurrence can be arduous, but the literature agrees that none of them have been uniquely associated to OBs in cattle, and their combined role is the most accredited hypothesis [41].

The Tuscany region was particularly affected by bTB, having the 50% of the OBs (*n* = 11) only in the last year, in the face of the previous five years (since 2018) in which only one or two OBs per year were observed. Moreover, this FT is associated with more cases of still open OBs, as well as with a considerable percentage (54.5%) of OB detection at the slaughterhouse.

In the following section, we provide a focus on the possible epidemiologically relevant primary determinants of the OB, based on our deeper knowledge of this region and the observations from the epidemiological investigation conducted by the local CA to determine the source of the OB.

### 3.4. Epidemiologically Relevant Primary Determinants of OBs in the Tuscany Region

The presence of OBs, especially in the last year, could be linked to a co-presence of factors. It is opportune to underline that many of the OBs observed in 2022–2023 were from a precise area located within the Florence province. The epidemiological investigation that was conducted by the local CA into the infection has provided some indications. First, in this area, all of the herds are semi-extensive, with animals housed indoors during autumn/winter and grazing in spring/summer. The pastures often consist of company-owned land, in which there are fences made of a single electric wire that separate the grazing areas of adjacent farms. This type of fencing can result in the movement of animals (e.g., calves) from one field to another. Also, it was observed that in nearby farms there was an exchange of equipment for harvesting silage and straw. In fact, it has been observed by the local CA that OBs in one farm occurred following OBs in the adjacent/nearby farm. The interactions between cattle on nearby or linked premises (e.g., exchange of equipment or adjacent fields) is in fact reported in the literature as a possible epidemiological link for transmission of bTB [42,43]. These were actually indicated by Magnani et al. [16] as causes of the detected OBs in three herds in the Emilia-Romagna region. The epidemiological investigation performed by the CA also highlighted that some OBs could be linked to the cattle movements from incidence areas, as on occasions of animal trade exchanges or animal fairs. In this respect, OBs have been often correlated with cattle movements [17,44,45]. In a study conducted in Castilla y Leon (Spain), it was actually observed that bTB-positive herds had a significantly higher number of moved cattle than negative herds [45]. Cattle movements could well be a route of bTB transmission because of the difficulty in detecting the infection (clinical signs are rare, and non-specific and diagnostic tests are imperfect) [45,46].

In Italy, a report on the epidemiological situation and surveillance activities in the Marche region (updated to December 2011) highlighted that most of the OBs reported in previous years were the result of introducing infected cattle from a certain stable in Apulia [47]. Also, Amato et al. [30] conducted an epidemiological investigation into a dairy cattle farm in the Veneto region infected by *M. caprae* and reported that some cattle testing positive originated from Austria; these animals were then temporarily relocated to a lairage facility in the Trento province (Trentino-Alto Adige region). A precise legislation imposes that the introduction of cattle to an FT is allowed only if they come from MTBC-free establishments. The movements within the national territory of cattle from non-FTs are regulated by the Agreement of 28 April 2022 of the Permanent Conference for Relations between the State, the Regions, and the Autonomous Provinces of Trento and Bolzano [34]. The local veterinary service, territorially competent, is required to prepare an annual list of establishments authorised to move cattle from non-FTs to FT areas. This list, validated by the Regional Authority, includes establishments meeting specific conditions as outlined in the Agreement, such as correct animal identification, uninterrupted MTBC-free status for the previous two years, and other criteria. Before the movement (exclusively to fattening farms) to an FT, specific pre-movement checks must be performed [34]. Throughout the national territory, the owner of the animals must individually register each beast in the national database of livestock (Banca Dati Nazionale dell’Anagrafe Zootecnica—BDN) within seven days of identification and in any case before each movement of electronically identified animals [27]. According to the same legislation, in the case of suspected animal substitution, alteration of identification, or unauthorised movement, the CA suspends the MTBC-free status of the affected establishment [27]. The illicit trade of infected cattle with counterfeit ear tags is reported in Italy [48]. Therefore, maintaining (or enforcing) cattle traceability systems is undoubtedly crucial for tracking the spread of bTB, as well as the study of the network of potential transmission routes based on interactions between herds [45].

Another interesting aspect that has emerged during the epidemiological investigations performed by the local CA is dated to 2024, which is after the period to which our data refers. In this year, suspected bTB macroscopic lesions were observed in the carcasses of some wild boars during postmortem examination: no such suspected lesions were found in any of the examined wild boar carcasses of the previous years. The laboratory analyses are currently in progress to confirm the hypothesis of bTB in these animals. Contact with wildlife can be regarded as an important factor driving the spread of bTB infection [2,8,18,49,50]. Indeed, bTB is difficult to eradicate in cattle particularly where it has become endemic in a wildlife population [51,52]. The role of wildlife in the epidemiology of bTB varies according to different epidemiological contexts: they can be only spill-over hosts or bTB sentinels of environmental mycobacterial contamination, maintenance hosts where infection can persist without an external source, or even super-shedders excreting significantly higher amounts of tuberculous bacilli than standard shedders [53]. This was also confirmed molecularly, with *M. bovis* strains found to be common to both wild and domestic animal populations [49,52,54]. Molecular confirmation is also reported in studies conducted in Italy. For instance, the same *M. bovis* strain isolated in wild boars was identified in a free-range cattle herd in the Marche region [55]. In Italy, the bTB prevalence in wild boar was reported to be 23.84% [2] and the significance of this species as an indicator for the presence of bTB in grazing cattle has been known for a long time [55]. According to the most recent data of the Italian Institute for Environmental Protection and Research (Istituto Superiore per la Protezione e la Ricerca Ambientale—ISPRA), the national population of wild boars has increased from approximately 500,000 individuals in 2010 to more than one million in 2020 (www.isprambiente.gov.it; accessed on 13 July 2024). It seems that 30% of the wild boar in the entire country is concentrated in Tuscany [56]. The population has progressively increased over the years, probably due to the increase in forested and uncultivated areas, the optimal climatic and habitat conditions, the increase in no-hunting areas, and the decrease in the number of hunters [57]. Moreover, a general conservative management approach aimed at restoring ungulate populations in protected or private areas, with a lack of a global and forward-looking vision regarding the effects that introductions/reintroductions or new colonisation would subsequently have, should be highlighted [57].

In case the suspected lesions in Tuscany wild boars are confirmed to be bTB, it will not be possible to establish if the presence of the disease in wildlife originates from infected herds or vice versa. Certainly, it may thereafter spread between wildlife species and back to livestock [9]. In fact, contact with wildlife reservoirs and the presence of infected neighbouring farms are reported as the two most frequent causes of the bTB breakdowns in the study by Ciaravino et al. [42].

Understanding how bTB moves through wildlife communities is essential for managing OBs [9]. In countries such as Italy, diagnosing and monitoring bTB in wild boars is especially crucial in establishing comprehensive eradication schemes [53,58]. However, accurate and detailed population statistics, such as census and hunting data, are often not available, with a persistent lack of comprehensive information of interactions at the wildlife–livestock interface [2,58]. In addition, the diagnosis of bTB in free-ranging wildlife is undoubtedly more difficult than in domestic animals [53]. It is therefore necessary, in the fight against bTB as well as other infectious diseases, to establish a detailed and documented inspection activity for this species to measure the epidemiological trend of the disease. In particular, it is essential to have precise information on the annual population estimate data, as well as culling data with respective individual identification and estimation of age and sex, identification of game collection centres, and hunting lodges where postmortem examination of wild boars is conducted; moreover, it would also be beneficial to establish the definition of a postmortem examination protocol related to the sites of tubercular infection, creation of specific forms for sample submission to the laboratory, veterinary inspection data with related outcomes for each individual identified by the hunter, and results of anatomical and pathological examinations for each individually identified animal [55].

Finally, it is important to highlight that farmers are especially responsible for the sanitary status of introduced animals and for biosecurity measures in order to deal with risk factors that could lead to the herd’s contamination, such as wildlife–livestock interactions [2,18]. Therefore, they must be forewarned of any analysis of state-sponsored disease control or eradication efforts and, as also reported by EU legislation, be encouraged to acquire, maintain, and develop adequate knowledge of animal health through relevant programmes or formal education [19]. In the case of OB management by CA in the Tuscany region, an active participation of farmers was observed, which is essential in order to limit under-reporting by the non-notification of suspected cases [59,60].

## 4. Conclusions

Bovine tuberculosis is an important disease that has been re-emerging in different ecological scenarios. In addition to its economic importance for the livestock industry, its zoonotic nature deserves the greatest attention as a public health risk. In Italy, despite the eradication programmes in force, OBs continue to be reported in FTs, suggesting the endemic condition of this disease. By analysing the official data available for the OBs in the FTs, a slight (but not statistically significant) decrease in the OB incidence rate after the FSY was observed, suggesting that bTB continues to be endemic. A notable decrease in OBs detected at the herd level (especially by official serological tests) and a corresponding increase in OBs detected at the slaughterhouses after the FSY were observed. This implies that infected animals may have evaded the control system during breeding (serological tests), highlighting issues in regional surveillance and eradication plans.

Differences in the herd control frequency (i.e., the percentage of herds that must be annually tested serologically to maintain the MTBC-free status of the FT) specific for each FT according to its regional plan of control and surveillance of bTB seems to not have a significant influence on the observed OB number in the FT. Overall, OBs may be linked to a co-presence of factors.

In the Tuscany region, the local CA, who performed an epidemiological investigation due to the increase in territorial OBs, observed that the main epidemiologically relevant primary determinants were the farming system (semi-extensive and adjacent herds) and the cattle movements from incidence areas (trade and animal fairs). The role of wildlife, especially wild boars, in disease maintenance cannot be excluded based on the evidence revealed by epidemiological investigations.

Overall, this study confirmed that it is crucial to maintain and enforce the national eradication programmes, adjusting the regional plans based on their risk assessments, including redefining regional surveillance and establishing coordinated, shared, and effective actions to manage OBs. The farmers’ implementation of biosecurity measures, especially for preventing contacts between cattle of different herds and between cattle and wildlife, can be considered one of the most effective measures to control the risk of bTB OBs. Also, it is particularly critical to establish a constant dialogue between the farm veterinarians and the official veterinarians, from which, in cases where the risk assessment has highlighted grave issues, a “customised enhanced biosecurity plan” can emerge. This would serve as a valuable complement to the existing regulatory framework, which, although effective, cannot be perfect due to the intrinsic and unavoidable limitations of the diagnostic tools on which it relies. The maintenance of the MTBC-free status ensures that farms benefit from health, economic, and commercial advantages, reducing the frequency of inspections, leading to savings in human and material resources for the National Health System.

## Figures and Tables

**Table 1:** MTBC-free territories (FTs) investigated in this study with relative declaration of free status year (FSY) and acquisition process.

FT (Region)	Area	MTBC-Free Status Declaration	
		FSY	Process
Trentino-Alto Adige	NE	Before 2003	autonomous provinces of Trento and Bolzano separated
Friuli-Venezia Giulia	NE	2006	Direct acquisition
Emilia-Romagna	NE	2007	Direct acquisition
Veneto	NE	2008	BL, PD (2007)
Lombardy	NW	2010	BG, LC, SO (2003), CO (2005)
Tuscany	Central	2010	GR (2004), LI, LU, SI (2007), PI, PT (2008)
Piedmont	NW	2016	NO, VB (2007), VC (2008), AT, BI (2012)
Liguria	NW	2016	Direct acquisition
Umbria	Central	2017	Direct acquisition
Valle d’Aosta	NW	2020	Direct acquisition
Molise	South	2021	Direct acquisition
Abruzzo	South	2022	PE (2006)
FT (province) *	Area	FSY	-
AP (Marche)	Central	2003	-
OR (Sardinia)	Islands	2009	-
CA, OG, OT #, vs. (Sardinia)	Islands	2010	-
RI, VT (Lazio)	Central	2011	-
AN, PU (Marche)	Central	2016	-
FR (Lazio)	Central	2018	-
MT (Basilicata)	Central	2021	-
SS (Sardinia) #	Islands	2021	-
LT (Lazio)	Central	2022	-
BA, TA (Apulia)	South	2022	-
NU (Sardinia)	Islands	2022	-
CZ (Calabria)	South	2023	-
NA (Campania)	South	2023	-
BT, BR, LE (Apulia)	South	2023	-
GS (Sardinia)	Islands	2023	-

* respective region is reported in brackets. # province was suppressed. AN: Ancona; AP: Ascoli Piceno; AT: Asti; BA: Bari; BR: Brindisi; BT: Barletta-Andria-Trani; BG: Bergamo; BI: Biella; BL: Belluno; CA: Cagliari; CO: Como; CZ: Catanzaro; FR: Frosinone; GR: Grosseto; GS: Gallura Nord Est Sardegna; LC: Lecco; LE: Lecce; LI: Livorno; LT: Latina; LU: Lucca; MT: Matera; NA: Napoli; NO: Novara; NU: Nuoro; OG: (Ogliastra); OR: Oristano; OT: Olbia-Tempio (suppressed); PD: Padova; PE: Pescara; PI: Pisa; PT: Pistoia; PU: Pesaro Urbino; RI: Rieti; SI: Siena; SO: Sondrio; SS: Sud Sardegna (suppressed) TA: Taranto; VB: Verbania; VC: Vercelli; VT: Viterbo; VS: Medio-Campidano.

**Table 2:** Herd control frequency (percentage of herds that must be annually tested serologically to maintain in the MTBC-free status) reported in the regional plans of control and surveillance of bTB of the MTBC-free territories (FTs) (of those available online). The number of controlled herds for each reported FT was estimated based on the number of herds reported in the same FT on 31 December 2023 (data from National Informative Veterinary System—NIVS (https://www.vetinfo.it/j6_statistiche/#/report-pbi/1, accessed on 3 June 2024).

FT Region	CF (%)	Number of Herds on 31 December 2023	Estimated Number of Controlled Herds in 2023
Abruzzo	100.0 *	3977	3977
Friuli-Venezia Giulia	33.3	1620	539
Lombardy	25.0	14,387	3596
Umbria	25.0	2980	745
Valle d’Aosta	25.0	1167	291
Liguria	20.0	955	191
Tuscany	20.0	3296	659
Veneto	20.0	11,330	2266
FT province	CF (%)	Number of herds on 31 December 2023	Number of controlled herds in 2023
Marche (AP, AN, PU)	50.0	2600	1300
Lazio (RI, VT, FR)	25.0	10,413	2603

* only for the first two years after the FSY (2022 for this region).

## Data Availability

Database of the National Informative Veterinary System (https://www.vetinfo.it/j6_statistiche/#/report-pbi/1); National Veterinary Epidemiological Bulletin (https://www.izs.it/BENV_NEW).

## Supplementary Materials

The following supporting information can be downloaded at: https://www.mdpi.com/article/10.3390/pathogens13110962/s1, Table S1: Italian territories declared MTBC-free with relative legislative source; Table S2: Information on number and year of bTB outbreaks (OBs) in each MTBC-free territory (FT); Territories with no OBs are not reported.
