# Supplementary material for: An Overview of a Re-Emerging Disease in Italy: Bovine Tuberculosis Outbreaks in Cattle from MTBC-Free Territories
Source: Pathogens. 2024 Nov 5;13(11):962. doi: 10.3390/pathogens13110962 (PMC11597750; doi:10.3390/pathogens13110962)
Supplement: Supplementary file 1 [file pathogens-13-00962-s001.zip › pathogens-3106360-supplementary.pdf]

Table S1. Italian territories declared MTBC-free with relative legislative source.

**Territories declared MTBC-free**	**Legislative source**
Region Trentino-Alto Adige: Province Bolzano, Province Trento	1999/467/CE
Region Marche: Ascoli Piceno<br>Region Lombardy: Bergamo, Lecco, Sondrio	2003/467/CE
Region Tuscany: Grosseto	2004/230/CE
Region Lombardy: Como<br>Region Tuscany: Prato	2005/28/CE
Region Abruzzo: Pescara	2006/169/CE
Region Friuli-Venezia Giulia	2006/290/CE
Region Emilia-Romagna<br>Region Piedmont: Novara, Verbania<br>Region Tuscany: Livorno, Lucca, Siena<br>Region Veneto: Belluno, Padova	2007/174/CE
Region Piedmont: Vercelli<br>Region Tuscany: Pisa, Pistoia	2008/97/CE
Region Veneto	2008/404/CE
Region Sardinia: Oristano	2009/342/CE
Region Lombardy<br>Region Tuscany<br>Region Sardinia: Cagliari, Medio-Campidano, Ogliastra, Olbia-Tempio	2010/391/CE
Region Lazio: Rieti, Viterbo	2011/277/UE
Region Piedmont: Asti, Biella	2012/204/UE
Region Liguria<br>Region Piedmont<br>Region Marche: Pesaro-Urbino, Ancona	2016/168/UE
Region Umbria	2017/888/UE
Region Lazio: Frosinone	2018/1983/UE
Region Valle d’Aosta	2020/552/UE
Region Molise<br>Region Basilicata: Matera<br>Region Sardinia: Sud Sardegna<br>(modifica province Sardegna dal 2016)	2021/385/UE
Region Abruzzo<br>Region Lazio: Latina<br>Region Apulia: Bari, Taranto<br>Regione Sardinia: Nuoro	2022/1218/UE
Regione Calabria: Catanzaro<br>Regione Campania: Napoli<br>Regione Apulia: Barletta-Andria-Trani, Brindisi, Lecce<br>Regione Sardinia: Nord-Est Sardegna	2023/1071/UE

Table S2. Information on number and year bTB outbreaks in each MTBC free-territory (FT); Territories with no outbreaks are not reported.

FT	Overall outbreaks (n)	outbreaks after MTBC-free status (n)	%	Years with bTB outbreaks (with outbreaks number)
RI (Lazio)	47	47	100	2011 (2); 2012 (2); 2013 (4); 2014 (9); 2015 (4); 2016 (1); 2017 (6); 2018 (3); 2019 (3); 2020 (4); 2021 (2); 2022 (2); 2023 (5)
CZ (Calabria)	36	0	0.0	-
Molise	35	5	14.3	2022 (4); 2023 (1)
Abruzzo	28	2	7.1	2022 (1); 2023 (1)
Veneto	25	25	100	2010 (3); 2011 (1); 2013 (16); 2015 (1); 2017 (2); 2018 (1); 2019 (1)
Piedmont	24	8	33.3	2015 (5); 2021 (1); 2022 (2)
Tuscany	22	22	100	2012 (1); 2013 (1); 2014 (1); 2018 (1); 2019 (1); 2020 (1); 2021 (1); 2022 (2); 2023 (11)
NA (Campania)	21	0	0.0	-
Lombardy	19	19	100	2013 (4); 2014 (1); 2015 (2); 2016 (1); 2017 (1); 2018 (3); 2019 (3); 2022 (3); 2023 (1)
FR (Lazio)	18	10	55.5	2018 (2); 2019 (1); 2020 (2); 2021 (1); 2022 (1); 2023 (3)
LE (Apulia)	14	0	0.0	-
BA (Apulia)	11	1	9.1	2023 (1)
NU (Sardinia)	11	1	9.1	2022 (1)
LT (Lazio)	10	0	0.0	-
Umbria	9	4	44.4	2012 (3); 2022 (1)
Emilia-Romagna	8	8	100	2011 (1); 2012 (1); 2018 (2); 2019 (2); 2020 (1); 2022 (1)
AN (Marche)	4	4	100	2023 (4)
MT (Basilicata)	4	0	0.0	-
TA (Apulia)	4	1	25.0	2022 (1)
BT (Apulia)	4	0	0.0	-
Friuli-Venezia Giulia	3	3	100	2014 (1); 2015 (1); 2019 (1)
Liguria	3	0	0.0	-
AP (Marche)	2	2	100	2020 (1); 2021 (1)
VT (Lazio)	2	2	100	2013 (1); 2014 (1)
Valle d'Aosta	2	0	0.0	-
SS (Sardinia)	2	1	50.0	2023 (1)
OR (Sardinia)	1	1	100	2021 (1)
BR (Apulia)	1	0	0.0	-
TOTAL	370	166	44.9	X
